# Difference of genomic copy numbers alterations between hairy cell leukemia-variant and classical hairy cell leukemia: a pilot retrospective study in Chinese

**DOI:** 10.7150/ijms.39307

**Published:** 2020-01-18

**Authors:** Rui Zhang, Yongli Wu, Xianfu Wang, Xianglan Lu, Yan Li, Shibo Li, Xiaojing Yan

**Affiliations:** 1 Department of Hematology, The First Affiliated Hospital of China Medical University, Shenyang, Liaoning, 110001, P.R. China.; 2 Department of Pediatrics, University of Oklahoma Health Sciences Center, Oklahoma City, OK 73104, USA.

**Keywords:** genomic copy numbers, hairy cell leukemia-variant, classical hairy cell leukemia

## Abstract

**Background:** Hairy cell leukemia (HCL) is a rare chronic B-cell lymphoproliferative disorder. It has two pathological subtypes: classical HCL (HCL-C) and HCL-variant (HCL-V). HCL-C and HCL-V are distinct in morphology and immunophenotype. Their differentiation is important for patient management and clinical outcome, with HCL-V responding poorly to conventional HCL treatments. Recently, whole genomic sequencing has been used to identify the difference between HCL-C and HCL-V and mutation of BRAF^V600E^ has been proved to be a molecular hallmark of HCL-C. However, BRAF inhibitors were not effective in all HCL-C cases and HCL-V seems be lack of the high-frequency mutations. Therefore, it is necessary to compare the genomic changes between HCL-C and HCL-V by high-resolution studies, especially in Chinese population, the genomic alterations of HCL have rarely be reported.

**Methods:** In this study, the clinical features of a total of 18 Chinese HCL patients were described. Single nucleotide polymorphism (SNP) array analysis was performed to evaluate the genomic copy number alterations (CNA) and copy neutral loss of heterozygosity (CN-LOH) on six HCL-Vs with CD25^-^/BRAF^V600E-^ and four HCL-Cs with CD25^+^/BRAF^V600E+^.

**Results:** A total of 24 CNAs including seven chromosomal gains and 17 chromosomal losses, and 22 CN-LOHs were revealed. Five of the six cases of HCL-V showed 15 CNAs including four cryptic chromosomal gains and 11 chromosomal losses. Overlapping regions involving micro-deletion of chromosome 2q13 and large chromosomal loss of 14q were showed in HCL-V. In HCL-C, a total of nine CNAs were revealed in three of the four cases including three chromosomal gains and six chromosomal losses. No overlapping area was observed among the CNVs. 15 CN-LOHs were showed in five of the six cases of HCL-V and seven CN-LOHs was demonstrated in all of the four HCL-Cs.

**Conclusions:** Comparing to Westerners, a relatively higher proportion of HCL-V in all HCL is observed in this study. CNAs and CN-LOHs were common in both HCL-V and HCL-C but the CNAs were different in them. HCL-C was characterized with the higher ratio of large chromosomal changes but lacked of recurrent CNAs, while HCL-V was presented with the higher incidence of cryptic CNAs and recurrent CNAs involving tumor-associated genes. It is necessary to further investigate the association of the genes, such as *NPHP1* and *TRAF3* genes, and HCL-V in the future study.

## Introduction

Hairy cell leukemia (HCL) is an uncommon chronic B-cell lymphoproliferative disorder that is characterized by aggregation of clonal small mature B lymphocytes with “hairy” projections on the cell surface in peripheral blood, bone marrow and spleen. Since HCL-variant(HCL-V) was first reported by Cawley *et al*. in 1980, the indolent form of classical HCL (HCL-C) and the more aggressive HCL-V (accounting for 10% of all HCL) has been included in HCL reporting for at least two decades (1-2). HCL-C and HCL-V share some common pathological features with each other but also differ in the hairy cell morphology, immunophenotype, response to conventional HCL treatments and disease courses 3-5. In 2008, HCL-V was included in the World Health Organization (WHO) classification as a provisional entity and is no longer considered to be biologically related to HCL-C 6. Therefore, identification of biological differences is essential to illuminate the underlying distinctions between HCL-V and HCL-C.

Current research in the field of HCL is focused on identifying novel and potential genomic aberrations which serve as specific diagnostic markers and therapeutic targets. Whole-exome sequencing made a great advancement in this area. In 2011, the activating* BRAF*^V600E^ mutation was found in all 47 patients with HCL 7. Further studies not only confirmed this finding but also identified that it was absent in both HCL-V and HCL-C accompanied with the IGHV4-34 molecular variant 8-9. Now, the *BRAF*^V600E^mutation has been a molecular hallmark and a treatment target of HCL-C 10-11. Recently, a high prevalence of *MAP2K1* mutation was observed in HCL-V and VH4-34^+^-HCL but not in HCL-C 12. However, other forms of genomic aberrations still need to be clarified because the activating *MAP2K1* mutation only occur in 50% of HCL-V and BRAF inhibitors were not effective in all HCL cases^13^.

HCL lacks balanced chromosomal abnormalities. Moreover, traditional banding analysis is frequently inconclusive due to chromosome condensation, imperfect banding and the presence of just a few metaphases. Genome-wide single-nucleotide polymorphism (SNP) arrays containing hundreds of thousands of SNPs from the human genome have proven useful for studying hematologic malignancies 14. Data quality of SNP arrays plays a key role in the accuracy and precision of unbalanced genomic aberrations and copy neutral loss of heterozygosity (CN-LOH) analyses. SNP array-based karyotyping was used in this study to identify the unbalanced chromosomal alterations in HCL, especially those cryptic alterations which are less than 5Mb in size and invisible in traditional G-banding.

## Methods

### Patients

Patients with HCL admitted to the First Affiliated Hospital of China Medical University from January of 2013 to December of 2016 were investigated in this study. All patients underwent bone marrow aspiration and biopsy. Referring to the 2008 World Health Organization (WHO) classification and previous studies, the diagnostic criteria of HCL-C or HCL-V was made: (1) lymphoid cells with hairy projections involving peripheral blood or bone marrow, (2) the pattern of interstitial or patchy infiltration in bone marrow biopsies,(3) the expression of B cell surface markers of (CD20) and the four markers (CD11c, CD25, CD103 and CD123) define the HCL score and (4) excluding other B cell hematological disorders^6^, ^13^,^15^-^16^.

### Morphology examination

Blood examination for all patients included complete blood counts and peripheral blood smears. Hairy cell morphology was described by bone marrow smear stains and bone marrow biopsies.

### Immunophenotype

Hairy cell phenotypes were determined by flow cytometry analysis of leukemic cells in bone marrow or peripheral blood. The expression panel of cell surface markers included CD19, CD20, CD22, CD23, CD25, CD11c, CD103, CD123, CD5 and FMC7 and κ/λ light chain classes were also determined.

### Single nucleotide polymorphism (SNP) array

DNA was extracted from bone marrow or peripheral blood mononuclear cells using the QiaAmp DNA Blood Minikit (Qiagen, Hilden, Germany). The DNA copy number and SNP analyses were performed with CytoScan® Array (n= 5) from Affymetrix® Protocols supplied by the manufacturer were followed. The Affymetrix®450 fluidics station and GeneChip® Scanner 3000 7G were used to wash, stain and scan the arrays.

SNP array data were analyzed using annotations of genome version GRCh37 (hg19) and were also compared to the Gene Expression Omnibus (GEO) database (accession number GSE67460). Detailed visual data analysis was performed in all samples, in addition to software-reported alterations. Only copy number aberrations (CNAs) with a gain more than 400 Kb, a loss of more than 200Kb and a LOH more than 100Kb and a minimum of 10 aberrant probes were selected. Detected CNAs were compared to the Database of Genomic Variants (http://dgv.tcag.ca/dgv/app/home) and an internal control series (n=1,000) provided by Affymetrix®in order to distinguish the somatic origin of CNAs from constitutional copy number variants (CNVs).

### *BRAF*^V600E^ mutation analysis

The mutation status of BRAF was assayed using Sanger sequencing in YuanQi Biomedical Technology Company (Shanghai, China). DNA was extracted from bone marrow or peripheral blood with DNeasy Blood & Tissue Kit (Qiagen, Inc.) for Sanger sequencing by Leukemia Related Gene Test Kit (Shanghai Yuanqi Bio‑Pharmaceutical Co., Ltd.). PCR reactions were carried out in a final volume of 25 μl containing 3 μl genomic DNA, 9μl sequencing reaction, and 13μl PCR MIX3 (Shanghai Yuanqi Bio‑Pharmaceutical Co., Ltd.). Samples were processed at 42˚C for 5 min and at 94˚C for 5 min, followed by 40 cycles at 94˚C for 30 sec, 58˚C for 30 sec, and 72˚C for 60 sec, with a final step for 5 min at 72˚C. PCR products were loaded on agarose gels, purified using BigDye Terminators and sequenced on ABI 3500 Genetic Analyzer.

## Results

### Epidemiology and clinical features

A total of 18 patients including 12 HCL-C and 6 HCL-V were diagnosed in the First Affiliated Hospital of China Medical University from 2013 to 2016. These 18 cases accounted for 1.9% of all the adult leukemia (acute myeloid /lymphoblastic leukemia and chronic myeloid leukemia, n=967) cases and 4.8% of all the lymphoma (non-Hodgkin's and Hodgkin's lymphoma, n=376) cases diagnosed at this hospital during this time span. The median age at diagnosis was 59.5 years (37-87 years old). The ratio of male to female was 8:1. Fever and fatigue were the most common presentations and splenomegaly was frequently observed on physical examination. The comparisons of clinical features between HCL-C and HCL-V are summarized in Table 1.

### Morphology of hairy cells

Of the 18 patients, 13 of them showed hypercellular bone marrow and three showed hypocellular bone marrow. Failure to collect bone marrow from two patients occurred due to dry tap. The median ratio of hairy cells was 55.5% ranged from 30.8% to 91.4% in peripheral blood or bone marrow. The cells were round in shape and varied in size from small to medium. The cytoplasm was abundant with irregular villiform projections. The nuclei were round, oval or reniform with a clear nuclear membrane and coarse reticular chromatin. Bone marrow biopsies from six patients showed lymphoid cells were spread out diffusely with abundant and clear cytoplasm which made wide spaces between the nuclei. Bone marrow fibrosis was observed in the two patients who had dry tap.

### Immunophenotype

All of the patients showed mature B lymphocyte immunophenotype with strong expression of CD19, CD20, CD22 and CD11c and restrictive expression of κ or λ light chains. The cells were also positive for FMC7 (n=14) and CD103 (n=15). All patients with HCL-C had CD25 expression, which was not presented in the six patients with HCL-V. The CD5 marker was negative in all patients except one (case #1 in HCL-C group) who had positive expression of CD103, FMC7 and CD25. None demonstrated positive expression of CD10. The characteristics of immunophenotype were summarized in Table 2.

### Genomic aberrations

**Figure 1:**
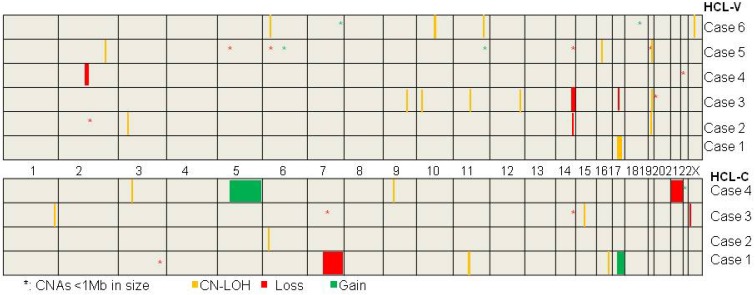
The delineation of genomic CNAs and CN-LOH in six HCL-Vs and four HCL-Cs by SNP-array analysis.

The mutation status of BRAF gene was assayed in ten cases. BRAFV600E mutation was demonstrated in four cases of HCL-C (case #7, 8, 9, 10) and wild type of BRAF was proved in all of the six HCL-Vs. SNP array analysis was performed on the ten cases and a total of 24 chromosomal number variations (CNVs) including seven chromosomal gains and 17 chromosomal losses, and 22 copy neutral loss of heterozygosity (CN-LOH) were revealed (Table 3 and Figure 1). There were no overlapping CNV regions between the HCL-V and the HCL-C. In HCL-V, a total of 15 CNAs was identified in five of the six cases. All of the four chromosomal gains were cryptic, involving chromosome 6, 11, 7 and 18. The majority of the 11 chromosomal losses were micro-deletions (<1Mb in size, n=7), whereas losses in large size (>5Mb in size, n=3) was not rare. Overlapping regions were showed as micro-deletion of chromosomes 2q13 (2q13:110,477,792-111,392,259, 0.914Mb) encompassing some interesting genes such as *NPHP1*, *RGPD6* and *LIMS4* and large chromosomal loss of 14q (14q32.13q32.33:96,207,358-105,637,747, 9.430Mb). In HCL-C, a total of nine CNAs were revealed in three of the four cases. Large chromosomal changes were common, including two of the three chromosomal gains and two of the six chromosomal losses. No overlapping area was observed among the CNVs. 15 CN-LOHs were showed in five of the six cases of HCL-V and seven CN-LOHs was demonstrated in all of the four HCL-Cs. The ratio of CN-LOH to CNV was 1:1 in HCL-V and 7:9 in HCL-C. The median size of CN-LOH was 1.988Mb (1.173-26.776Mb) in HCL-V and 2.653Mb (1.946-7.983Mb) in HCL-C. CN-LOH of 19q13.2 (42,471,566-43,371,294, 0.900Mb) was the overlapping region observed in HCL-V, containing interesting genes such as POU2F2, DEDD2, GSK3A, ERF, CIC, CXCL17, CEACAM1 and PSG. Overlapping area of CN-LOH was absence in HCL-C.

## Discussion

The term HCL is derived from observations that the clonal expansion of mature B cell had hair-like villi on the cell surface and frequently infiltrated the bone marrow, spleen and liver. Currently, HCL is classified by the WHO under B-cell non-Hodgkin's lymphoma 17. HCL rarely occurs in either leukemia or lymphoma. A ten-year study from the United States of America showed that HCL accounted for 4.4% of all adult leukemia and 1.1% of all lymphoma cases 18. In our study, the incidence of HCL in leukemia was lower than that in America, but it was higher in lymphoma. Incidency of HCL-V was higher, constituting 33.3% of all HCL cases in this study, while the proportion was 10% in Western countries 19. A similar prevalence was also observed in Taiwan, which demonstrated that the HCL-V occurs more frequently in Asian populations, while HCL-C is rare 20.

Clinically, it is difficult to differentiate HCL-C and HCL-V as they share many common presentations such as fever, anemia, splenomegaly and enlargement of lymph nodes. Immunophenotypes can help distinguish HCL-V from HCL-C with the typical markers panel 21-22. In our study, differentiation between HCL-V and HCL-C was made using the marker panel with CD23, CD123, CD25 and CD103. The specific molecular hallmark is the BRAF^V600E^ mutation which is observed in almost all patients of HCL-C but negative in other B-cell malignancies including HCL-V 23-24. As a serine/threonine kinase, BRAF is a member of the RAF family and involved in the mitogen-associated protein kinase (MAPK) signaling pathway 25-26. Therefore, BRAF is not only a disease-defining marker of HCL-C but is also a therapeutic target for BRAF inhibitors. In contrast, the molecular landmark of HCL-V is not known. Recently, activating mutations in the *MAP2K1* gene were reported in ten of twenty-four HCL-V cases and in five of seven HCL-C cases that were IGHV4-34^+^ by means of whole exome sequencing 12. However, another study confirmed that the *MAP2K1* mutation in HCL-V was not as high as 42% and it was also observed in splenic B-cell lymphoma/leukemia (unclassifiable) (SBCLL-U) 27. This indicates that sequencing analysis is not enough to delineate the genetic network of HCL.

Chromosomal copy number aberrations (CNAs) are common in lymphoid malignancies, but conventional cytogenetic analysis is frequently limited by tumor cell culture. In recent years, gene chip technology has been used to determine genomic unbalanced changes in leukemia cells 28-29. However, its application in HCL is limited, especially in Asian countries which have unique clinicopathologic features of HCLs 20. The diagnosis of HCL-V and HCL-C in this study was confirmed by typical morphology, immunophenotype as well as BRAF^V600E^ mutation status. SNP array analysis was applied on HCL-Vs with CD25^-^/BRAF^V600E-^ and HCL-Cs with CD25^+^/BRAF^V600E+^. The high incidence of CNA as well as CN-LOH in both HCL-V and HCL-C cases in this cohort of Chinese patients was consistent with the study of UK reported by Hockley *et al*. (2011) which identified the CNAs in 11 cases (86%) of HCL-C and 14 cases (93%) of HCL-V 30. However, Rinaldi and his colleagues (2013) showed that the incidence of CNAs in HCL-C was extremely low; only two genomic losses were detected in 19 cases of HCL-C 31. They concluded that the presence of genetic damages was low in HCL-C due to the high recurrence of the V600E inducing deregulated activity of the *BRAF* gene. Moreover, this study presented distinct genomic aberrations, in contrast with the study from UK which showed gain of 5q, deletion of 7q and deletion of 17p (including *TP53*) were recurrent aberrations in HCL^30^. It was assumed that gain of 5q and deletion of 7q were resulted from the TP53-related genomic instability since they coexisted with 17p-. However, in this study, 5q+ and 7q- were not recurrent CNAs and found respectively in two cases which showed no concurrent 17p- involving TP53 (Table 4).

In addition, distinct CNAs and CN-LOH changes were presented in HCL-V and HCL-C. It was characterized with the chromosomal loss and cryptic chromosomal alterations which were undetectable with traditional cytogenetic approaches. Especially, the ratio of large chromosomal change was higher in HCL-C, while HCL-V was more frequently contained cryptic CNAs. Moreover, HCL-V carried recurrent chromosomal abnormalities such as micro-deletion of chromosomes 2q13 and large chromosomal loss of 14q. *NPHP1*, locating at 2q13, complete deletion of this gene might cause nephronophthisis 32. The contribution to tumorigenesis of this gene is not known. However, since this gene encodes a protein with a SRC homology 3 (SH3) domain, which plays a role in cell division as well as in cell-cell and cell-matrix adhesion signaling, the association between *NPHP1* and HCL-V was worth investigating. The recurrent large chromosomal deletion of 14q32.13q32.33 in this study partially overlaps with an area in del(14)(q24.1q32.33) which is the recurrent chromosomal aberration in CLL and B cell lymphoma 33. One of the cases of HCL-V in Hockley's study also has been revealed to contain a microdeletion of 14q32.32^30^. A key tumor suppressor gene, *TRAF3*, is located within the deleted region. The protein encoded by this gene is a member of the TNF receptor associated factor (TRAF) protein family which is a critical component of the lymphotoxin-βreceptor (LTβR) signaling complex. This complex induces nuclear factor kappa B (NF-kB) and cell death is initiated by LTβligation^34^-^35^.

In conclusion, this study showed a higher incidence of HCL-V in the cohort of Chinese patients with HCL. The delineation of the genomic CNAs and CN-LOHs demonstrated the difference between HCL-V and HCL-C at the molecular level. The HCL-C showed higher ratio of large chromosomal changes but lacked of recurrent CNAs, on one hand supporting the driver mutation role of BRAF^V600E^ mutation, on the other hand suggesting that the effectiveness of BRAF inhibitors might not be observed in all HCL-C. The higher incidence of CNAs in HCL-V cases in this study suggests a higher heterogeneity in HCL-V. The recurrent micro-deletion of chromosomes 2q13 and large chromosomal loss of 14q32 shed light on the potential target of HCL-V such as *NPHP1* and *TRAF3* genes. It is necessary to clarify the association of these genes and HCL in the future study. Differentiation between HCL-C and HCL-V at the molecular target level could be important for identifying potential agents to improve results with HCL-V.

## Figures and Tables

**Table 1:** Clinical features of patients with HCL

	HCL-C	HCL-V
Total number	12	6
Median Age (range)	61 (38-87)	59 (37-69)
Male/Female	11/1	5/1
Fever	6	1
Fatigue	4	1
Splenomegaly	12	6
Lymphadenopathy	4	2
Pancytopenia	7	0
PB count median (range)		
WBC (×10^9^/L)	3.38 (1.33~128.54)	59.6 (14.39~223.16)
Hb (g/L)	88 (53~137)	133 (91~167)
Platelet (×10^9^/L)	62 (35~105)	122 (81~193)
Hairy cell(%)	46.95 (30.8 ~89.5)	66.05 (36.4~91.4)

PB: Peripheral Blood; WBC: White blood cell; Hb: hemoglobin

**Table 2:** The immunophentypes of 6 cases of HCL-V and 12 cases of HCL-C

Case No.	CD11c	CD25	CD103	CD123	CD23	FMC7	CD5
**HCL-C (n=12)**							
1	++	+	+	—	—	++	++
2	++	+	++	/	—	++	—
3	++	+	++	++	—	++	—
4	++	+	++	++	—	++	—
5	++	++	++	—	—	++	—
6	+	+	++	/	—	—	—
7	++	+	++	++	+	+	—
8	++	++	++	/	—	++	—
9	++	++	++	—	++	++	—
10	++	++	++	++	—	++	—
11	++	+	++	/	++	—	—
12	++	++	+	+	—	—	—
**HCL-V ( n=6)**							
1	++	—	—	—	—	++	—
2	++	—	+	/	—	+	—
3	++	—	+	—	+	++	—
4	++	—	++	—	—	—	—
5	++	—	—	—	—	+	—
6	++	—	—	—	—	++	—

++: strong expression, +: dim expression, —: negative, /: unknown

**Table 3:** Genomic aberrations identified by single nucleotide polymorphism (SNP) array analysis

	Case	Chromosomal aberration	Chromosome	Genomic Annotation (bp) GRCh37	Size (Mb)
**HCL-V (n=6)**	# 1	CN-LOH	17q22q25.3	54,265,339-81,041,823	26.776
	# 2	Loss	2q13	110,477,792-111,392,259	0.914
		Loss	14q32.13q32.33	96,207,358-105,637,747	9.430
		CN-LOH	3p21.31	48,186,267- 50,174,572	1.988
		CN-LOH	19q13.2	42,198,558-43,371,294	1.173
	# 3	Loss	14q32.13q32.33	96,182,616-106,237,059	10.054
		Loss	17q11.2	27,902,488-31,078,344	3.176
		Loss	20p12.1	15,101,464-15,207,464	0.106
		CN-LOH	9q31.1	103,873,786-105,991,203	2.117
		CN-LOH	10p14p13	10,249,027-13,875,590	3.627
		CN-LOH	11q13.1q13.2	65,399,528-66,842,469	1.443
		CN-LOH	12q24.31	121,944,131-124,325,360	2.381
		CN-LOH	19q13.2q13.31	42,471,566-43,807,152	1.336
	# 4	Loss	2q11.2q13	102,236,088-113,269,612	11.033
		Loss	22q11.22	22,724,607-23,121,204	0.397
	# 5	Loss	5q11.2	55,509,576-56,265,992	0.756
		Loss	6p21.33	31,360,255-31,453,640	0.093
		Gain	6q16.1	94,567,726-95,262,849	0.695
		Gain	11q23.3	117,954,250-118,038,741	0.084
		Loss	14q32.33	106,391,073-107,216,306	0.825
		Loss	19q13.12	37,285,393-37,469,098	0.184
		CN-LOH	2q32.1q32.2	188,962,426-190,835,473	1.873
		CN-LOH	16p11.2	29,912,902-31,411,185	1.498
		CN-LOH	19q13.2q13.31	42,390,241-43,695,840	1.306
	# 6	Gain	7q36.2	153,397,733-153,670,448	0.273
		Gain	18q21.2q21.31	53,630,341-53,899,387	0.269
		CN-LOH	6p22.2	26,089,334-27,491,299	1.402
		CN-LOH	10p11.21q11.21	37,737,079-44,015,746	6.279
		CN-LOH	11q22.2 q22.3	102,495,998-104,924,246	2.428
		CN-LOH	Xq13.1q13.3	71,586,774-75,087,152	3.500
**HCL-C (n=v)**	# 1	Loss	3q27.1	183,660,585-184,626,909	0.966
		Loss	7q11.21q11.22	66,377,234-159,114,952	92.738
		Gain	17q11.2q25.3	26,125,918-81,046,413	54.920
		CN-LOH	11p11.2q12.1	48,102,867-56,086,147	7.983
		CN-LOH	16q21q22.1	66,242,376-69,516,168	3.274
	# 2	CN-LOH	6p22.2p22.1	25,622,212-27,670,917	2.049
	# 3	Loss	7q21.11	80,040,648-80,211,718	0.171
		Loss	14q32.33	106,467,544-106,803,397	0. 336
		Loss	Xp22.31	6,456,940-8,135,053	1.678
		CN-LOH	1q43	239,313,573-242,808,496	3.495
		CN-LOH	15q15.1q21.1	42,437,431-45,090,821	2.653
	#4	Gain	5q11.1q35.2	49,708,187-173,216,595	123.508
		Loss	Monosomy 21		
		Gain	22q11.23q12.1	25,687,568-25,910,879	0.223
		CN-LOH	3p21.31	47,608,097-49,554,285	1.946
		CN-LOH	9q31.1	105,323,892-107,571,241	2.247

**Table 4:** Comparison of genomic CNAs between this study and other published studies

Author/Year	HCL-C*				HCL-V			
	Aberration	Loss	Gain	LOH	Aberration	Loss	Gain	LOH
Hockley *et al*./2011	NO.	28	19	21	NO.	81	37	57
	Recurrent**	7q, 21q	12p	-	Recurrent	3p, 6q, 7q, 8p, 14q, 17p, 19p,	5, 5q, 12q, 17q	-
Rinaldi *et al.*/2013	NO.	2	0	0	NO.	-	-	-
	Recurrent	0	0	-	Recurrent	-	-	-
Zhang *et al.*/The present study	NO.	6	3	7	NO.	11	4	15
	Recurrent	0	0	-	Recurrent	2q, 14q	0	-

*cases with HCL-C or HCL with BRAF^V600E+^ **Chromosomes with overlapping regions of deletion/gain occurring in ≥ 2 cases
